# Chemopreventive Agents from Nature: A Review of Apigenin, Rosmarinic Acid, and Thymoquinone

**DOI:** 10.3390/cimb46070393

**Published:** 2024-06-27

**Authors:** Reem Fawaz Abutayeh, Maram Altah, Amani Mehdawi, Israa Al-Ataby, Adel Ardakani

**Affiliations:** 1 Faculty of Pharmacy, Applied Science Private University, Amman 11937, Jordan; israa.adnan1985@yahoo.com; 2 School of Pharmacy, Al-Qadisiyah College, Amman 11118, Jordan; amanimehdawi@aqc.edu.jo; 3 College of Pharmacy, Amman Arab University, Amman 11953, Jordan; a.ardakani@aau.edu.jo

**Keywords:** natural chemoprevention, anti-cancer phytochemicals, natural chemotherapy, apigenin, rosmarinic acid, thymoquinone

## Abstract

Cancer, a major challenge to global health and healthcare systems, requires the study of alternative and supportive treatments due to the limitations of conventional therapies. This review examines the chemopreventive potential of three natural compounds: rosmarinic acid, apigenin, and thymoquinone. Derived from various plants, these compounds have demonstrated promising chemopreventive properties in in vitro, in vivo, and in silico studies. Specifically, they have been shown to inhibit cancer cell growth, induce apoptosis, and modulate key signaling pathways involved in cancer progression. The aim of this review is to provide a comprehensive overview of the current research on these phytochemicals, elucidating their mechanisms of action, therapeutic efficacy, and potential as adjuncts to traditional cancer therapies. This information serves as a valuable resource for researchers and healthcare providers interested in expanding their knowledge within the field of alternative cancer therapies.

## 1. Introduction

In both industrialized and developing countries, cancer is considered a high-profile disease that damages the body’s systems. Conventional treatments such as surgery, chemotherapy, and radiotherapy are commonly employed but often come with severe side effects, potential recurrence, and/or treatment failure. As a result, there is a growing interest in complementary and alternative medicine (CAM) for cancer treatment and prevention. Natural products, including herbal and phytochemical compounds, are the main biology-based practices within CAM, and they have grown to be a multibillion-dollar industry worldwide. Their long history of use supports their adoption in health promotion and disease prevention and treatment [1].

Phytochemicals such as carotenoids, polyphenols, and flavonoids have been studied for their potential in cancer prevention and treatment. These compounds are present in plant-based foods, beverages, supplements, and traditional herbal remedies [1,2]. Scientists have been particularly interested in phytochemicals because they target the biological pathways in mammalian cells that are involved in inflammatory processes and cancer development. These pathways include control of the cell cycle, apoptosis, angiogenesis, and metastasis. Epidemiological studies suggest that regularly ingesting phytochemicals can reduce the incidence of various cancers.

Many phytochemicals, including assorted polyphenols, are rapidly eliminated from the human body. However, they remain promising chemopreventive and/or chemotherapeutic agents due to their ability to target multiple cancer cell mechanisms with minimal toxicity to normal cells. Phytochemicals can be administered alone or in combination with conventional chemotherapeutic agents to explore synergistic or enhanced cytotoxic effects. Such combination treatments are also investigated for their potential to overcome chemotherapy-induced resistance, reduce adverse effects, and improve the safety profile for normal cells [2].

Cancer chemoprevention involves the use of natural, synthetic, or biological chemical agents to reverse, inhibit, or prevent carcinogenic initiation or the progression of cancer. This approach is considered one of the most effective strategies to reduce the risk of cancer development and recurrence. Bioactive molecules used in chemoprevention can target different stages of carcinogenesis, acting as inhibitors of carcinogen formation, and interfering with the initiation or post-initiation steps to halt or reverse the development of premalignant cells [3,4,5].

Several interconnected mechanisms of chemoprevention include the modulation of phase I and phase II metabolic enzymes [6], oxidative stress, inflammatory pathways such as the COX-2 pathway, signal transduction, and hormones [7].

In this review, we summarize the mechanisms of three natural phytochemicals—rosmarinic acid, apigenin, and thymoquinone—with different chemical structures and derived from commonly used plants. Although numerous in vivo and in vitro studies support their chemopreventive potential, further research is needed to fully understand their mechanisms of action, to determine the most effective dosages and routes of administration, and to confirm their safety and efficacy in a clinical setting.

Despite their distinct chemical structures and mechanisms of action, these compounds share common bioactivities, such as antioxidant and anti-inflammatory properties, cancer growth inhibition, apoptosis promotion, and the modulation of cell proliferation and survival pathways [5,6,7].

## 2. Rosmarinic Acid (RA)

**Figure 1:**
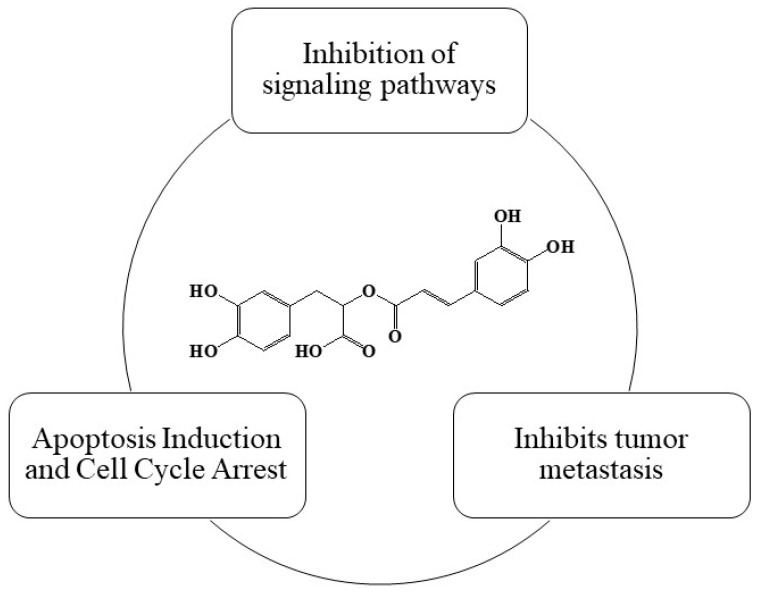
Summary of RA’s chemopreventive properties.

Rosmarinic acid (RA) is a polyphenolic acid found in various herbs and plants, including rosemary, sage, lemon balm, marjoram, thyme, and oregano. The richest sources of RA are members of the Lamiaceae family. RA exhibits antioxidant and anti-inflammatory properties and can inhibit the growth of cancer cells in vitro. It has been shown to suppress the expression of genes involved in cancer cell proliferation and metastasis, as well as inducing apoptosis through the modulation of signaling pathways related to cell survival and death. A summary of RA’s chemopreventive properties is shown in Figure 1.

### 2.1. Source and Chemical Structure

**Figure 2:**
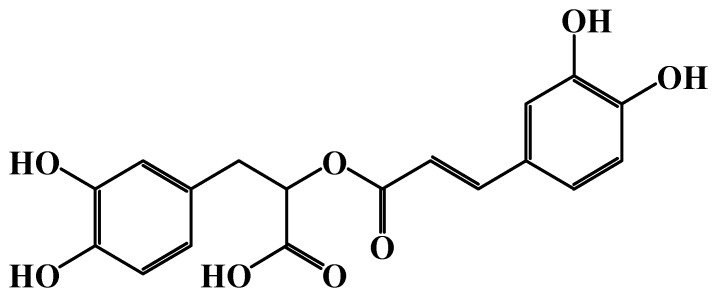
Chemical structure of RA (ChemDraw Ultra 7.0).

RA (C_18_H_16_O_8_), an ester of caffeic acid and 3,4-dihydroxy phenyl lactic acid (Figure 2), is considered one of the most important polyphenolic compounds present in nature [8,9].

Although RA is widespread throughout the plant kingdom, it was first isolated and characterized in 1958 from rosemary (*Rosmarinus officinalis*, Family: Lamiaceae) and named after it. However, rosemary is not considered the primary source or the richest source of RA [9,10]. For example, a high-performance liquid chromatographic analysis of 29 plants belonging to the Labiatae family revealed that Mentha species had the highest RA content, with a considerable concentration ranging from 19.3 to 58.5 mg/g compared to that found in rosemary (7.2 mg/g) [11].

### 2.2. In Vivo and In Vitro Studies of RA

#### 2.2.1. In Vitro Studies of RA

Given its strong antioxidant properties, RA has been extensively investigated for its chemopreventive potential, and consequently, additional mechanisms of action have been proposed. Yang et al. (2021) reported that RA inhibited tumor metastasis in the human colorectal cancer cell line HT-29, and its effect was mediated primarily by suppressing epithelial–mesenchymal transition [12]. RA also restricted apoptosis and tumor growth in hepatic cancer-induced nude mice in a dose-dependent manner. The inhibitory activity of RA was further illustrated to take place via the PI3K/AKT/mTOR signaling pathway in SMMC-7721 hepatocellular carcinoma cell lines [13]. Moreover, RA demonstrated chemotherapeutic potential in OVCAR-3 ovarian cancer cells, causing cellular shrinkage, apoptosis, and cell migration suppression in a time- and concentration-dependent manner at 10, 40, and 160 μM concentrations after 48 and 72 h [14].

In triple-negative breast cancer cell lines (MDA-MB-231 and MDA-MB-468), RA induced apoptosis and cell cycle arrest by altering the transcription of multiple apoptosis-related genes in both cell lines [15]. RA also inhibited glucose uptake and lactate production in human gastric cancer (MKN45) cell lines in vitro, demonstrating an anti-Warburg effect at concentrations up to 600 μM, and suppressed gastric tumor growth in a mouse xenograft model treated with 2 mg/kg of RA for 14 weeks [16].

#### 2.2.2. In Vivo Studies of RA

Despite its notable cytotoxic effects in vitro, RA extracts have been less extensively studied in vivo in animal cancer models. The available studies (shown in Table 1) have not fully elucidated the mechanisms underlying RA’s chemopreventive effects. Furtado et al. (2015) investigated RA’s anti-carcinogenic capability and showed that RA, orally administered at doses up to 16 mg/kg/day, successfully reduced the extent and frequency of DNA damage and tumor formation in rats treated with the colon carcinogen 1,2-dimethylhydrazine (DMH) at a dosage of 40 mg/kg [17]. Likewise, when RA was orally administered at a dose of 5 mg/kg for 30 weeks, it prevented tumor formation and induced apoptosis in DMH-induced rats. RA prevented colon cancer by inducing pro-apoptotic protein expression [18]. Additionally, an oral dose of 100 mg/kg/day of RA was administered to a skin cancer animal model for a week. RA prevented tumor formation and induced apoptosis in Swiss albino mice treated with dimethylbenz(a)anthracene (DMBA) [19].

RA’s chemopreventive mechanism of action was further investigated using colorectal cancer animal models. Colorectal cancer was induced in male BALB/c mice and then was treated with a 30 mg/kg/day oral dose of RA for a week, and it was found to suppress tumor progression in mice through the inhibition of TLR4-mediated NF-κB and STAT3 activation. This mechanism was also proven in vitro using the HCT116 colorectal carcinoma cell line, where RA competitively inhibited the TLR4-MD-2 complex [20].

RA enhanced tumor sensitivity to several chemotherapeutic drugs, such as cisplatin and doxorubicin, which are common chemotherapeutic agents capable of inducing multi-drug-resistant (MDR) gene expression. When RA and doxorubicin were given in combination in a breast cancer mouse model, the combination exhibited better pharmacokinetics and anti-cancer efficacy than doxorubicin alone [21]. Also, RA enhanced the apoptotic effect of doxorubicin by activating the mitochondria-mediated signaling pathway in HepG2 and Bel-7402 cell lines [22]. In another study, the efficacy of RA was tested in cisplatin-resistant NSCLC lung cancer cells both in vitro and in xenograft tumors in nude mice. The combination therapy resulted in the significant downregulation of MDR1 mRNA and P-GP expression in vitro and a marked inhibition of NSCLC xenograft tumor growth in nude mice [23]. Similarly, the inhibition of renal cancer cell invasion and migration using cisplatin was augmented when combined with RA [24].

Regarding RA’s apparent toxicity in vivo, Xue et al. (2021) reported that the body weight of mice did not show obvious changes during RA treatment. Furthermore, no systemic toxicity was observed in histopathological examination of the major organs, including the heart, liver, spleen, lungs, and kidneys [21].

**Table 1:** Summary of in vivo studies of rosmarinic acid (RA).

Study Focus	Animal Model	RA Dose and Route of Administration	Findings	Reference
Prevention of colon cancer induced by 1,2-dimethylhydrazine (DMH)	Rats	Oral, up to 16 mg/kg/day	RA significantly reduced DNA damage and tumor formation	[17]
Prevention of colon cancer induced by DMH	Rats	Oral at 5 mg/kg/day for 30 weeks	RA prevented tumor formation and induced apoptosis	[18]
Prevention of skin cancer induced by DMBA	Swiss Albino mice	Oral at 100 mg/kg/day for a week	RA prevented skin cancer and induced apoptosis	[19]
Mechanisms of RA anti-cancer activity in colorectal cancer	Male BALB/c mice	30 mg/kg/day for a week	RA suppressed tumor progression by inhibiting TLR4-mediated NF-κB and STAT3 activation	[20]
Enhancement of doxorubicin’s efficacy in breast cancer	Female BALB/C mice	IV 8 mg/kg combined with doxorubicin	Combination showed better pharmacokinetics and anti-cancer efficacy than doxorubicin alone	[21]
Enhancement of cisplatin’s efficacy in cisplatin-resistant NSCLC lung cancer	Xenograft in nude female BALB mice	Intraperitoneal, combined with cisplatinvolume of administration of 10 μL/g	Significant inhibition of tumor growth with cisplatin combined with RA when compared to cisplatin alone	[23]

### 2.3. In Silico Studies of RA

The effect of RA on cell lines has been extensively studied, showing its ability to induce apoptosis in cancer cells—a critical mechanism for cancer treatment. Although many modeling studies are found for RA, they mainly address its activity in other fields of pharmacological activities, such as its antiviral, antimicrobial, and anti-diabetic potential activities, among others. Chemoprevention-related in silico studies remain limited. Anwar et al. (2020) studied the activity of RA’s anti-cancer effect in relation to microtubule affinity regulating kinase (MARK4) inhibition. RA demonstrated excellent binding affinity to the active site and formed several hydrogen bonds with critical residues, which led to MARK4 inhibition and apoptosis induction [25]. Jelić et al. (2007) studied the modes of RA binding to the Fyn tyrosine kinase and concluded that it binds to the non-ATP binding site of the kinase. The Fyn tyrosine kinase is an Src-family enzyme involved in T-cell receptor signal transduction and was found to be inhibited experimentally by RA. This study combined experimental and computational methods in order to understand the mechanism of RA chemoprevention activity [26].

RA’s ability to induce apoptosis and inhibit cancer cell growth by regulating signaling pathways makes it a promising candidate for further development as an anti-cancer drug. However, more studies are needed to fully understand the mechanisms of its anti-cancer effects and to optimize its therapeutic potential. In vitro and in vivo studies of RA used various concentrations of RA and different cancer cell lines or animal models to investigate the underlying mechanisms of RA’s chemoprevention effect.

## 3. Apigenin

**Figure 3:**
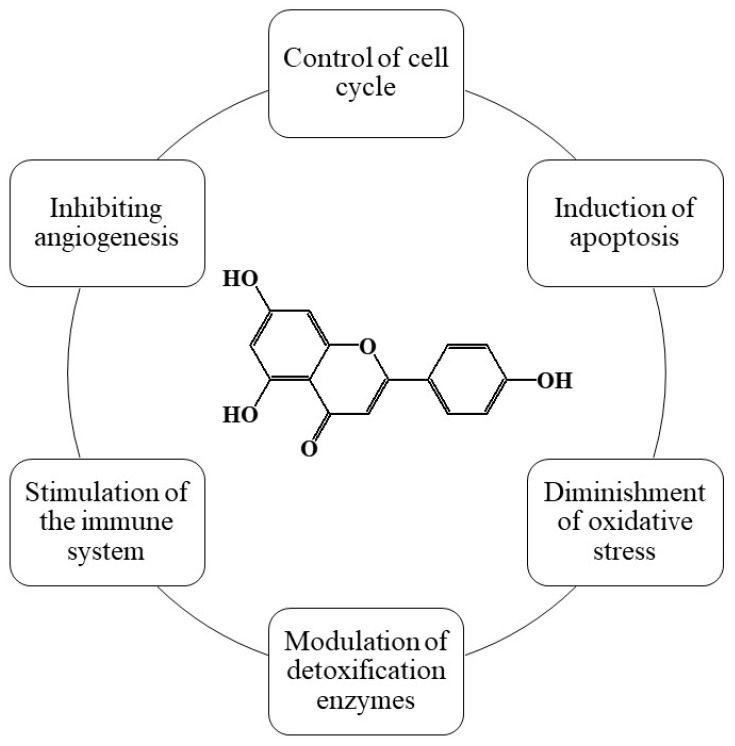
Summary of apigenin’s chemopreventive properties.

Apigenin is a flavonoid that is found in a variety of plants, including parsley, celery, and chamomile. It has been shown to have antioxidant, anti-inflammatory, and anti-cancer properties. In vitro studies have demonstrated that apigenin inhibits the growth of cancer cells, induces cell cycle arrest, and promotes apoptosis. Additionally, apigenin has been shown to modulate signaling pathways involved in cell proliferation and survival and to inhibit angiogenesis and metastasis. A summary of apigenin’s chemopreventive properties is shown in Figure 3

### 3.1. Source and Chemical Structure

**Figure 4:**
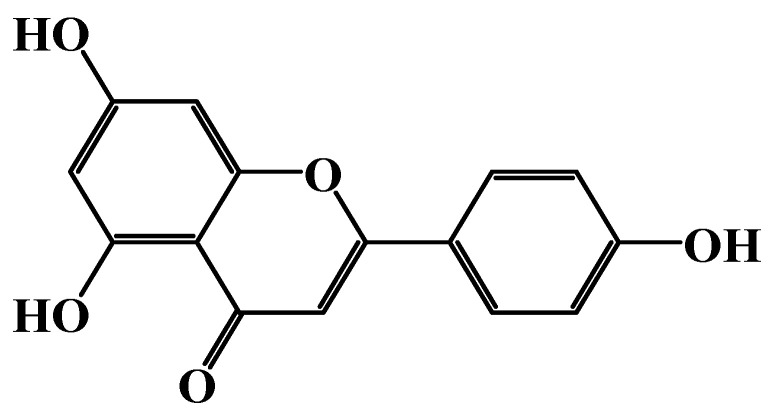
Chemical structure of apigenin (ChemDraw Ultra 7.0).

Apigenin (4′, 5, 7-trihydroxyflavone; C_15_H_10_O_5_) is a flavonoid with a molecular weight of 270.24 g/mol. Its chemical structure is shown in Figure 4. Apigenin is mainly found in parsley, which is scientifically named *Petroselinum crispum*, from the family Umbelliferae (also called Apiaceae) [27,28].

It is also found in different quantities in a variety of other plants, including celery, basil, chamomile, and many others. Table 2 presents common sources of apigenin along with the approximate quantities found in each.

### 3.2. In Vitro and In Vivo Studies of Apigenin

#### 3.2.1. In Vitro Studies

In vitro studies have been pivotal in illuminating the mechanisms through which apigenin exerts its anti-cancer effects. These studies have demonstrated that apigenin can inhibit the proliferation of various cancer cell lines, including prostate [31,32,33,34,35,36], breast [37,38,39,40,41], colon [42,43], and lung cancers [44,45]. The mechanisms involved include the modulation of cell cycle regulatory proteins [46,47], the inhibition of angiogenesis [48,49], and the modulation of key signaling pathways, such as the PI3K/AKT/mTOR [50] and PI3K/Akt/FoxO 3a pathways [35,51], which are crucial for cancer cell survival and proliferation.

Apigenin reduces the viability of cancer cells in a dose-dependent manner, where in an MTT assay, apigenin treatment significantly decreased the viability of human breast cancer MCF-7 cells, indicating its potential to selectively target cancerous cells [52]. It exerts its anti-cancer effects through the induction of apoptosis in various cancer cell lines and through multiple pathways. It triggers the activation of caspases, which are essential executors of apoptosis. Studies have demonstrated that it increases the activity of caspase-3 and caspase-9, leading to the cleavage of poly (ADP-ribose) polymerase (PARP), a hallmark of apoptosis. Moreover, it disrupts mitochondrial membrane potential, leading to the release of cytochrome c into the cytosol, which subsequently activates the apoptotic machinery. This mitochondrial pathway is crucial to the intrinsic apoptosis pathway [53].

Furthermore, apigenin alters the expression of the Bcl-2-family proteins, which are key regulators of apoptosis. It downregulates anti-apoptotic proteins like Bcl-2 and Bcl-xL while upregulating pro-apoptotic proteins such as Bax, promoting apoptosis in cancer cells [33].

Apigenin also induces DNA damage in cancer cells, leading to cell cycle arrest and apoptosis. This is evidenced by the increased expression of γ-H2AX, a marker of DNA double-strand breaks, following apigenin treatment [54].

Overall, these in vitro studies underscore the potential of apigenin as a chemopreventive agent by demonstrating its ability to reduce cell viability and induce apoptosis in cancer cells through various molecular mechanisms, including the modulation of signaling pathways, cell proliferation, apoptosis, inflammation, and angiogenesis.

#### 3.2.2. In Vivo Studies

In vivo studies examined the effect of the dietary consumption of apigenin (0.1%) in the prevention of cancer in azoxymethane-induced colon rat models. Apigenin triggered the apoptosis of luminal surface colonocytes, reduced the incidence of aberrant crypt foci, and decreased peritoneal metastasis incidence [55]. Additionally, dietary intake of apigenin (0.2%) for six weeks in nude mice with A549 lung cancer xenografts reduced tumor volume, attributed to the suppression of the HIF-1α–vascular endothelial growth factor pathway [56].

One notable in vivo study used a transgenic mouse prostate adenocarcinoma (TRAMP) model, where TRAMP mice were given 20 and 50 μg/mouse of apigenin orally for 20 weeks. This study revealed that apigenin reduced tumor volumes and distant organ metastasis, attributed to the suppression of the PI3K/Akt/Forkhead box O-signaling pathway [35].

Furthermore, oral administration of apigenin (2.5 mg/kg) in hamsters with DMBA-induced oral cancer for 15 weeks reduced tumor volume and incidence and modulated markers of cell proliferation, apoptosis, inflammation, and angiogenesis [57,58].

Oral administration of apigenin (3 mg/kg) in nude mice bearing human lung cancer xenografts decreased the tumor volume and wet weight, reduced serum IGF-I levels, and induced apoptosis and cell cycle arrest [59]. Additionally, oral administration of apigenin in Apc^Min/+^ mice contributed to a reduction in polyp numbers through the activation of p53 [60].

Topical application of apigenin (5 and 20 μmol) in murine skin tumorigenesis, initiated by DMBA and promoted by TPA in SENCAR mice, resulted in a marked reduction in the incidence and number of papillomas and carcinomas [61].

In UVB-induced skin inflammation in SKH-1 hairless mice, topical application of apigenin (5 μM) prior to UVB exposure reduced UVB-induced ear edema and COX-2 expression, modulated HIF-1α, and suppressed mTOR signaling [62].

As for its toxicity, apigenin was found to have no apparent in vivo toxicity in xenograft tumor models, including regular mice [32], BALB/c mice [54], and athymic nude mice [44]. The safety of apigenin was demonstrated at doses of 30 mg/kg and 10 μg/mouse intraperitoneally for 21 days [44,54], as well as at doses of 20 and 50 μg/mouse/day for up to 56 days [32]. Its non-toxicity was assessed by monitoring body weight changes [32,44], blood cell count differences [54], and liver mass and histological variations [44] compared to control groups, respectively. A summary of apigenin’s in vivo studies are shown in Table 3.

### 3.3. In Silico Studies of Apigenin

Apigenin was effectively docked with the cellular tumor antigen p53, caspase-3, and the mucosal addressin cell adhesion molecule 1, exhibiting significant interactions based on their calculated binding energies [63]. Another in silico study using molecular dynamics simulation evaluated apigenin’s binding propensity towards class I histone deacetylase (HDAC) isoforms. The study found apigenin to have a high binding affinity for most of the class I HDACs, particularly demonstrating stability in the binding pocket of the HDAC2 isoform, with numerous contacts persisting for more than 30% of the simulation duration [64].

## 4. Thymoquinone (TQ)

**Figure 5:**
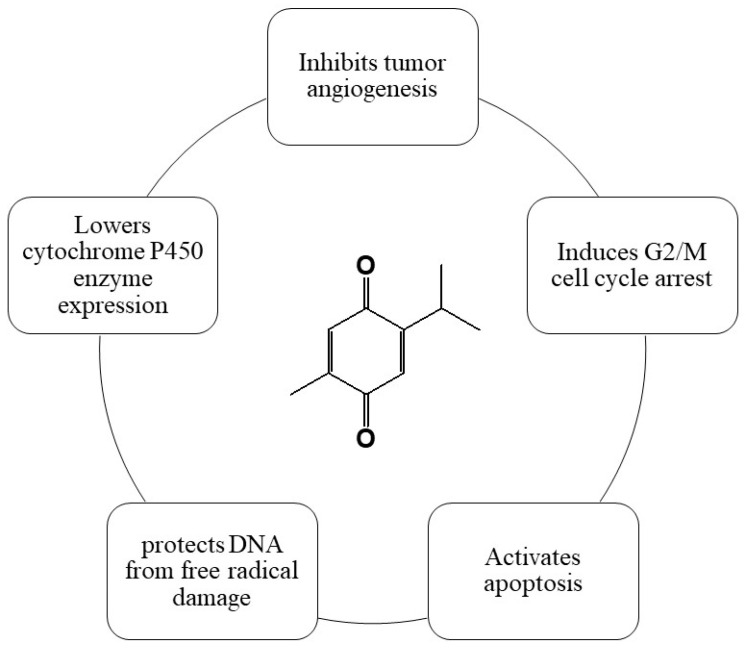
Summary of TQ’s chemopreventive properties.

TQ has antioxidant, anti-inflammatory, immunomodulatory, anti-histaminic, anti-microbial, and anti-tumor action, making it a promising natural chemopreventive agent [53,54,55,56]. A summary of its chemopreventive properties are shown in Figure 5.

### 4.1. Source and Chemical Structure

**Figure 6:**
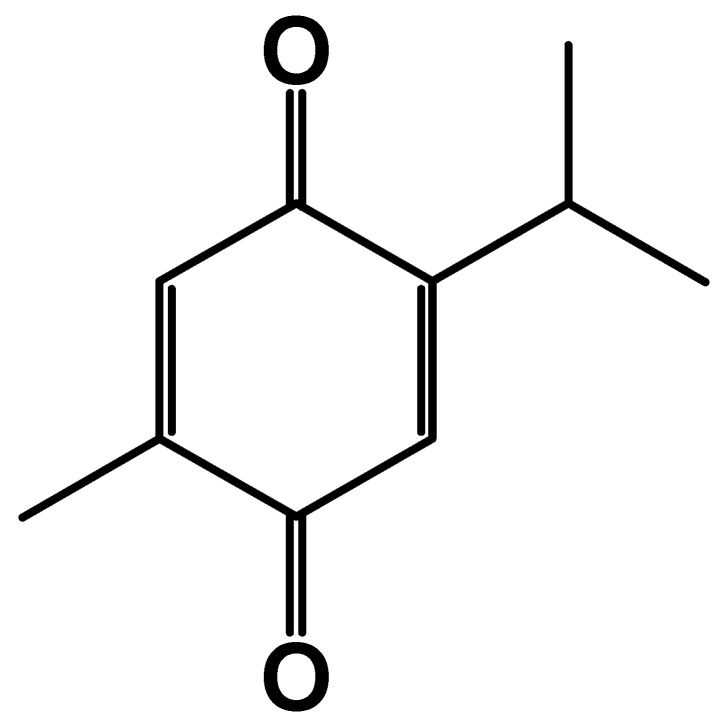
Chemical structure of TQ (ChemDraw Ultra 7.0).

Thymoquinone (TQ) is the main bioactive terpene constituent (Figure 6) found in the volatile oil isolated from *Nigella sativa* (black cumin, black seed), which has been used as a traditional medicine in many countries [52,53]. It can also be found in other plants, as shown in Table 4.

### 4.2. In Vivo and In Vitro Studies of TQ

#### 4.2.1. In Vitro Studies of TQ

TQ has been shown to inhibit the proliferation of various cancer cell lines, including those from colon and colorectal [65,66,67], breast [68,69,70], pancreatic [71], and other types of cancer [72,73]. This inhibition is often mediated through the modulation of cell cycle regulatory proteins. TQ can induce cell cycle arrest at different phases, primarily G1/S and G2/M, by regulating cyclins and cyclin-dependent kinases (CDKs) [74].

In addition, TQ induces apoptosis by activating both intrinsic and extrinsic apoptotic pathways, increasing the expression of pro-apoptotic proteins such as Bax and p53, while decreasing the levels of anti-apoptotic proteins like Bcl-2 [75,76]. TQ also induces the activation of caspases, which are crucial executors of apoptosis [77].

Furthermore, TQ exhibits potent anti-inflammatory effects by inhibiting the expression of pro-inflammatory cytokines such as TNF-α, IL-1β, and IL-6. It also suppresses the NF-κB signaling pathway, which is often upregulated in cancer and associated with inflammation and cell survival [78].

TQ has been shown to induce oxidative stress selectively in cancer cells, leading to cell death. It increases reactive oxygen species (ROS) generation, which can damage cellular components and induce apoptosis. Conversely, TQ enhances the antioxidant defense mechanisms in normal cells, thereby protecting them from oxidative damage [79,80].

Moreover, TQ inhibits key processes involved in cancer metastasis and angiogenesis. It downregulates the expression of matrix metalloproteinases (MMPs), enzymes that degrade the extracellular matrix and facilitate metastasis. Additionally, TQ reduces the expression of vascular endothelial growth factor (VEGF), thereby inhibiting angiogenesis and restricting tumor growth [81,82].

In vitro studies utilizing MCF-7 breast cancer cells have shown that TQ induces cell cycle arrest at the G1 phase and promotes apoptosis through the upregulation of p21 and p27 and the downregulation of cyclin D1 [66]. Likewise, studies on PC-3 prostate cancer cells have demonstrated that TQ induces apoptosis through the mitochondrial pathway and inhibits cell proliferation by suppressing the AKT signaling pathway [79,82]. In addition, studies with HCT-116 colon cancer cells have found that TQ treatment results in significant inhibition of cell proliferation and the induction of apoptosis via the modulation of the Wnt/β-catenin signaling pathway [79,83]. TQ has also been shown to sensitize pancreatic cancer cells to gemcitabine, a standard chemotherapeutic agent, by enhancing ROS production and downregulating NF-κB signaling, leading to increased apoptosis [84,85].

These in vitro cytotoxicity studies and their findings highlight the potential of TQ as a chemopreventive agent by demonstrating its ability to inhibit cell proliferation, induce apoptosis, exert anti-inflammatory effects, modulate oxidative stress, and inhibit metastasis and angiogenesis.

#### 4.2.2. In Vivo Studies of TQ

Numerous in vivo studies have examined TQ’s potential therapeutic effects, particularly in cancer treatment. A summary of in vivo studies of TQ are found in Table 5 These studies have highlighted TQ’s ability to inhibit tumor growth, metastasis, and angiogenesis across various cancer models, supporting further exploration of TQ as a therapeutic agent.

Several studies have investigated TQ’s effects on different types of cancer in animal models. In a study on Ehrlich acid solid tumors in mice, TQ was administered via intraperitoneal injection at a dose of 10 mg/kg for four weeks (five doses per week). The results showed that TQ reduced oxidative stress, prevented necrosis, and enhanced tissue regeneration [86].

In another study on thioacetamide-induced liver cancer in rats, TQ was given through oral gavage at a dose of 20 mg/kg body weight for 16 weeks. The findings indicated that TQ induced apoptosis by upregulating TRAIL and caspase-3, downregulated Bcl2 and TGF-β1, improved liver function, and reduced hepatocellular carcinoma progression [87].

In a study on mouse epithelial breast cancer cell line (EMT6/P) xenografts in BALB/C mice, the co-administration of TQ with piperine prevented tumor growth by decreasing VEGF expression and increasing serum INF-γ levels, leading to apoptosis [88].

Similarly, in a study on breast cancer cell lines in BALB/C mice, the co-administration of TQ with resveratrol induced apoptosis and decreased VEGF expression [89]. Furthermore, a study on breast cancer in mice, where TQ was administered orally at a dose of 65 mg/kg body weight, revealed that TQ reduced tumor markers, suppressed histopathological changes, and regulated the expression of Brca1, Brca2, P53, and Id-1 mutation [90].

Roepke et al. (2007) conducted a study on osteosarcoma (OS), using human osteosarcoma cell lines MG63 and MNNG/HOS in a xenograft mouse model. TQ was administered in vivo at a dose of 6 mg/kg/day, significantly reducing the expression of NF-κB protein in OS tumors [75].

Another study on human pancreatic ductal adenocarcinoma (PDAC) cells showed that TQ dose-dependently arrested the G2 cell cycle phase and reduced cell growth and viability, increased p53 and p21 expression, and decreased Bcl-2 expression, leading to tumor size reductions [91].

Additionally, Peng et al. (2013) studied SaOS-2 cells (human osteosarcoma) and found that TQ exhibited pro-apoptotic effects in a concentration-dependent manner, reducing cancer cell proliferation through various molecular pathways, including ROS generation and MAPK signaling. TQ also reduced the DNA-binding activity of NF-κB in a dose-dependent manner and significantly attenuated the expression of the NF-κB protein in osteosarcoma tumors [92].

**Table 5:** Summary of in vivo studies of TQ as a chemopreventive agent.

Cancer Type	Cell Lines	Animal Model	TQ Dosage	Mechanism of TQ Action	Overall Outcome	References
Bladder cancer	T-24 and 253 J cell lines	Xenograft mouse	10 mg/kg/3 days	↑ E-cadherin, ↓ N-cadherin, vimentin, Wnt/β-catenin, MYC, axin-2, MMP7, cyclin D1	Augmentation of gemcitabine anti-cancer activities through the upregulation of apoptosis and autophagy processes	[93]
Breast cancer	MDA-MB-231 and MDA-MB-436	Xenograft mouse	5 mg/kg/day	↑ miR-361, ↓ Rac, RhoA, VEGF-A	Angiogenesis and metastasis suppression and a decrease in tumor weight	[94]
Cervical cancer	SiHa cell lines	-	-	↑ p53, ↓ Bcl-2	Cell cycle arrest at the sub-G1 phase, induction of apoptosis and necrosis	[95]
Glioblastoma	S6 cell lines	-	-	↓ ERK, FAK, MMP-2, MMP-9	Reduced cell survival, migration, adhesion, and metastasis processes	[96]
Liver cancer	SNU-7721 and HepG2 cell lines	Xenograft rats	20 mg/kg/day	↑ Bax, caspase-8, ↓ Bcl-2, VEGF	Cell cycle arrest at G2/M phase and induction of apoptosis	[97]
Prostate cancer	DU-145, PC-3	Xenograft mouse	5–30 mg/kg/2 days	↑ E-cadherin, ↓ Slug, TGF-β, Smad-2, Smad-3, vimentin	Reduced cell survival, migration, and invasion	[98]
Prostate cancer	PC-3, LNCaP	Xenograft mouse	10–20 mg/kg/day	↑ p21, p27, caspases; ↓ Bcl-2, Cyclin D1, CDK4	Induction of apoptosis, cell cycle arrest, inhibition of tumor growth	[99]
Gastric cancer	BGC-823, HGC-27, MGC-803, SGC-7901	Xenograft mouse	20 mg/kg/day	↑ Bax, caspase-3, caspase-9, cytochrome c, ↓ Bcl-2	Increased sensitivity to 5-FU, induction of apoptosis, decrease in tumor weight	[100]
Gastric cancer	BGC-823, HGC-27, SGC-7901	Xenograft mouse	10–30 mg/kg/2 days	↑: Bax, caspase-3, caspase-7, caspase-9 ↓: Bcl-2, cyclin D, c-Src, JAK2, STAT3, survivin, VEGF	Inhibition of cell growth and angiogenesis, apoptosis induction, and reduction in tumor weight	[72]
	HGC-27, MGC-803, and SGC-7901	Xenograft mouse	10 mg/kg/2 days	↑: AIF, Bax, caspase-3, caspase-9, cytochrome c, PTEN ↓: Bcl-2, cyclin D1, p-gp	Increased sensitivity to cisplatin, induction of apoptosis, decrease in tumor weight	[101]
Colorectal cancer	HCT 116wt, DLD-1, HT29	-	25 mg/kg/day	↓ ERK1/2, MEK, PAK1	Decreased cell viability, induction of apoptosis and necrosis, decrease in tumor weight	[102]
Colorectal cancer	5FU-resistant HCT116	Xenograft mouse	20 mg/kg/2 days	↑ p21, p53, γH2AX, ↓ CD44, EpCAM, ki67, NF-κB, MEK	Induction of apoptosis, reduced cell invasion and migration, decrease in tumor weight	[103]
	Irinotecan (CPT-11)-resistant LoVo cell lines			↓: IKKα/β, NF-κB, Snail, Twist, vimentin, MMP-2, MMP-9, ERK1/2, PI3K	Increased cell rate, mitochondrial mem- brane permeability, induction of apoptosis and autophagy	[104]
Pancreatic cancer	PANC-1, BxPC-3	Xenograft mouse	5 mg/kg/day	↑ Bax, caspase-3, p53; ↓ Bcl-2, NF-κB	Induction of apoptosis, suppression of tumor growth	[105]
Lung cancer	A549	-	-	↓: cyclin D1, ERK1/2, MMP-2, MMP-9, PCNA	Decreased rate of cancer cell proliferation, migration, invasion, and metastasis, cell cycle arrest at the G0/ G1 phase	[106]
Lung cancer	A549	-	-	↓: Bcl-2	Decreased cell viability and induction of Apoptosis, as well as necrosis Depolymerization of microtubules and disruption of mitotic spindle organization, promotion of apoptosis, and decrease in cell viability	[107,108]
Ovarian cancer	ID8_NGL, NCI/ADR, and OVCAR-3	Xenograft mouse	20 mg/kg/2 days	↓: Bcl-2, PCNA	Increased cell death, sensitivity of cancer cells to cisplatin, induced apoptosis	[109]
Ovarian cancer	SK-OV-3 cell lines	-	-	↓: Bcl-2	Induced apoptosis, cell cycle arrest at the S phase, and reduced anti-cancer impact of cisplatin	[110]

↑ upregulate, ↓ downregulate.

### 4.3. In Silico Studies of TQ

A reverse in silico study was applied to TQ against a variety of targets involved in metastasis and apoptosis to search for potential targets involved in cancer therapy. The results found that TQ successfully binds to apoptotic targets such as TRAIL-R, Bcl-2, MDM2, Bak, Bax, and PARP [111]. TQ was docked to the active site of the target PTEN, which is a negative regulator of the PI3K/AKT pathway. TQ illustrated a great binding affinity to PTEN, with the potential to inhibit abnormal cell proliferation via modulating the activity of PTEN [112]. Furthermore, TQ’s position as a p53 activator and preventative anti-cancer agent against lung cancer is implicated by the docking data [113].

## 5. Comparison of RA, Apigenin, and TQ: Chemopreventive Mechanisms and Extraction Methods

In vitro studies have significantly advanced our understanding of the chemopreventive mechanisms of RA, apigenin, and TQ, revealing both common pathways and unique targets affected by each compound, as shown in Table 6. Apigenin inhibits cancer cell proliferation by modulating cell cycle regulatory proteins, inducing cell cycle arrest [47], and promoting apoptosis [37,114]. It affects key signaling pathways such as PI3K/AKT and MAPK, inhibits angiogenesis, and disrupts the mitochondrial membrane, leading to apoptosis [48]. TQ similarly induces cell cycle arrest and apoptosis but also prominently activates both intrinsic and extrinsic apoptotic pathways and modulates oxidative stress by increasing ROS generation selectively in cancer cells. Additionally, TQ has potent anti-inflammatory effects, inhibiting pro-inflammatory cytokines and the NF-κB signaling pathway, and suppresses metastasis and angiogenesis by downregulating MMPs and VEGF [85]. RA also shows potential in cancer prevention through its strong antioxidant properties and modulation of several signaling pathways, including PI3K/AKT/mTOR [12]. It induces apoptosis and cell cycle arrest in various cancer cells, suppresses metastasis, and exhibits anti-Warburg effects by inhibiting glucose uptake and lactate production [16]. Collectively, these compounds target cell proliferation, apoptosis, angiogenesis, and inflammatory pathways but with unique aspects of their mechanisms, such as RA’s anti-Warburg effect and TQ’s ROS-mediated cytotoxicity. Table 6 illustrates a concise comparison of the chemopreventive mechanisms for RA, apigenin, and TQ.

The interest in the various bioactivities of RA, apigenin, and TQ has led to the development of efficient methods of extraction from their natural sources. For RA, the methods include vibration, maceration with stirring, heat reflux, Soxhlet solvent extraction, and recent innovations like ultrasound-assisted, microwave-assisted, enzyme-assisted, and pressurized-liquid extraction, with the solvent choice playing a crucial role in yield optimization [115]. Apigenin extraction benefits from advanced techniques such as dynamic maceration and the use of ionic liquid analogs, specifically deep eutectic solvents (DESs), which offer efficient and environmentally friendly alternatives [116]. TQ extraction involves hydrodistillation (HD) using a Clevenger-type apparatus, dry steam distillation (SD), steam distillation of crude oils obtained by solvent extraction (SE-SD), and supercritical fluid extraction (SFE-SD), with CO2 as the preferred solvent due to its cost-effectiveness and safety [117]. These varied methods reflect ongoing efforts to optimize the extraction processes for these valuable phytochemicals.

## 6. Clinical Studies

Among the three natural compounds—rosmarinic acid (RA), apigenin, and thymoquinone (TQ)—there are no adequate clinical studies supporting their use as chemopreventive agents, despite promising data from in vivo and in vitro studies [115,116,117,118,119,120].

One clinical study involving apigenin, titled “Dietary Bioflavonoid Supplementation for the Prevention of Neoplasia Recurrence” (ClinicalTrials.gov Identifier: NCT00609310), aimed to evaluate the efficacy of bioflavonoid supplementation in reducing the recurrence of colorectal neoplasia. Conducted by Technische Universität Dresden, this phase 2 interventional study involved 382 participants aged 50 to 75 years who had recently undergone surgical resection for stage 2 or 3 colorectal cancer. It was designed as a double-blind, randomized, placebo-controlled trial, with the participants divided into two groups. The experimental group received a daily dietary supplement containing a mixture of bioflavonoids, specifically 20 mg of apigenin and 20 mg of epigallocatechin gallate (EGCG) from chamomile and green tea extracts, along with vitamins and folic acid. The primary objective was to determine whether this supplementation could decrease the rate of neoplasia recurrence over a three-year period. The secondary outcomes included overall survival, recurrence-free survival, and serum levels of the bioflavonoids. Despite its promising design, the study’s recruitment status was listed as suspended as of the last update in February 2012, with the reasons for suspension unspecified.

TQ was investigated for its long-term safety in healthy human subjects at a dosage of 200 mg/day for 90 days. Hematological and biochemical parameters, including kidney and liver function tests and lipid profiles, were examined alongside anthropometric measurements to monitor safety. The study participants did not exhibit any side effects, indicating that TQ was well tolerated over the studied period [119].

A recent clinical study involving TQ, titled “Clinical and Immunohistochemical Evaluation of Chemopreventive Effect of Thymoquinone on Oral Potentially Malignant Lesions” (ClinicalTrials.gov Identifier: NCT03208790), investigated the potential of TQ, derived from Nigella sativa, as a chemopreventive agent for oral potentially malignant lesions (OPMLs). Conducted by Cairo University, this randomized, controlled, parallel-group trial involved 48 participants aged 18 to 75 years with histologically and clinically confirmed OPMLs. The participants were divided into three groups: Group A received Nigella sativa buccal tablets containing 10 mg of TQ; Group B received Nigella sativa buccal tablets containing 5 mg of TQ; Group C received placebo buccal tablets. The primary goal was to assess the clinical response by measuring the dimensions of the lesions at baseline and after three months of treatment. The secondary outcomes included immunohistochemical evaluations for markers of cell proliferation (Ki-67) and apoptosis (caspase-3). While specific results from the study are not detailed in the available summaries, the trial aimed to determine whether TQ could reduce lesion size and affect molecular markers indicative of malignant transformation. The study concluded in March 2020, but detailed results have not been posted publicly. This study represents a significant step in exploring natural compounds like TQ for cancer chemoprevention, specifically in OPMLs, although further detailed results are necessary to confirm its efficacy and potential for clinical use. According to the database, the study completed phase 2, with the last update posted on 20 April 2021 [121,122,123].

## 7. Conclusions

In this review, we highlight the chemopreventive potential of rosmarinic acid (RA), apigenin, and thymoquinone (TQ). These compounds, derived from widely used plants, exhibit diverse chemical structures and functions, including their ability to act as antioxidants, cell signaling modulators, and inhibitors of cell proliferation. Extensive in vitro, in vivo, and in silico studies provide a robust foundation for their chemopreventive properties. However, transitioning these agents into clinical use necessitates further clinical, toxicological, and pharmacokinetic studies to confirm their safety and efficacy in humans. Addressing pharmacokinetics and bioavailability challenges through advanced formulations, drug delivery systems, and chemical optimization is essential. Computational methods such as computer-aided drug design (CADD) and molecular dynamics simulations can significantly reduce the research costs and expedite the development of more potent and more selective chemopreventive derivatives.

Continuous investigation into these natural products could lead to complementary or alternative cancer treatments with fewer adverse effects than conventional therapies. Thus, future research should focus on the following: (1) conducting well-designed clinical trials to establish effective dosages, formulations, and delivery methods; (2) investigating the anti-metastatic potential and synergistic effects of RA, apigenin, and TQ in combination with other chemotherapeutic agents; and (3) long-term safety and toxicology studies to assess the potential adverse effects of their chronic use.

By addressing these research objectives, the potential of chemopreventive agents like RA, apigenin, and TQ in cancer treatment can be fully realized, leading to improved patient outcomes and innovative therapeutic options.

## Figures and Tables

**Table 2:** Sources and quantities of apigenin.

Source	Quantity of Apigenin (mg/kg)	Reference
Chinese cabbage	187.0	[29]
Bell pepper	272.0	[29]
Garlic	217.0	[29]
Bilimbi fruit	458.0	[29]
French peas	176.0	[29]
Guava	579.0	[29]
Wolfberry leaves	547.0	[29]
Daun turi	39.5	[29]
Kadok	34.5	[29]
Celery seeds	786.5	[30]
Spinach	620	[30]
Parsley	450.4	[30]
Marjoram	44.0	[30]
Oregano	35.0	[30]
Sage	24.0	[30]
Chamomile	30–50	[30]
Rosemary	5.5	[30]
Pistachio	0.3	[30]

**Table 3:** Summary of in vivo studies of apigenin as a chemopreventive agent.

Study Model	Apigenin Dosage and Administration	Key Findings	Reference
Colon carcinogenesis in rats	Dietary intake of 0.1% apigenin	Triggered apoptosis of luminal surface colonocytes, reduced aberrant crypt foci, decreased peritoneal metastasis	[55]
Lung cancer xenografts in nude mice	Dietary intake of 0.2% apigenin for 6 weeks	Reduced tumor volume, suppressed HIF-1α-VEGF pathway	[56]
Prostate cancer in TRAMP mice	Oral administration of 20 and 50 μg/mice for 20 weeks	Reduced tumor volumes and distant organ metastasis by suppressing PI3K/Akt/FoxO pathway	[35]
DMBA-induced oral carcinogenesis in hamsters	Oral administration of 2.5 mg/kg for 15 weeks	Reduced tumor volume and incidence, modulated cell proliferation, apoptosis, inflammation, and angiogenesis markers	[57]
Lung cancer xenografts in nude mice	Oral administration of 3 mg/kg	Decreased tumor volume and wet weight, reduced serum IGF-I levels, induced apoptosis and cell cycle arrest	[59]
APCMin/+ mice model	Oral administration of apigenin	Reduction in polyp number by activation of p53	[60]
Murine skin tumorigenesis in SENCAR mice	Topical application of 5 and 20 μmol	Marked reduction in the incidence and number of papillomas and carcinomas	[61]
UVB-induced skin inflammation in SKH-1 mice	Topical application of 5 μM prior to UVB exposure	Reduced UVB-induced ear edema and COX-2 expression, modulated HIF-1α, and suppressed mTOR signaling	[62]

**Table 4:** Common sources and quantities of TQ.

Source	Quantity of TQ (mg/kg)	Reference
*Eupatorium cannabinum* L.	8	[51]
*Juniperus communis* L.	6 free TQ, 15 glycosidically bound TQ	[51]
*Monarda didyma* L.	3029	[51]
*Monarda didyma* L.	3425	[51]
*Monarda media* Willd.	2995	[51]
*Monarda menthifolia* Graham	1381	[51]
*Satureja hortensis* L.	217	[51]
*Satureja montana* L.	1052	[51]
*Thymus pulegioides* L.	233	[51]
*Thymus serpyllum* L.	233	[51]
*Thymus vulgaris* L.	300	[51]
*Nigella sativa* L.	1881	[51]

**Table 6:** Comparison of mechanisms and molecular targets of apigenin, RA, and TQ.

Compound	Key Mechanisms	Specific Targets	References
Apigenin	Inhibits proliferation, induces apoptosis, modulates cell cycle	Cell cycle regulatory proteins, PI3K/AKT, MAPK, caspases, Bcl-2 family, mitochondrial membrane potential	[33,53]
Rosmarinic Acid (RA)	Induces apoptosis, inhibits metastasis, affects glucose metabolism (anti-Warburg effect) [13,16]	PI3K/AKT/mTOR, epithelial–mesenchymal transition, apoptosis-related genes, glucose uptake, and lactate production [13,16]	[13,16]
Thymoquinone (TQ)	Induces cell cycle arrest and apoptosis, modulates oxidative stress, anti-inflammatory, inhibits metastasis and angiogenesis	Cyclins, CDKs, p53, Bcl-2, Bax, NF-κB, ROS, MMPs, VEGF	[69,75,77,78,89]
